# Pharmacodynamic Target Assessment and PK/PD Cutoff Determination for Gamithromycin Against *Streptococcus suis* in Piglets

**DOI:** 10.3389/fvets.2022.945632

**Published:** 2022-07-11

**Authors:** Rui-Ling Wang, Ping Liu, Xiao-Feng Chen, Xin Yao, Xiao-Ping Liao, Ya-Hong Liu, Jian Sun, Yu-Feng Zhou

**Affiliations:** ^1^National Risk Assessment Laboratory for Antimicrobial Resistance of Animal Original Bacteria, College of Veterinary Medicine, South China Agricultural University, Guangzhou, China; ^2^Guangdong Provincial Key Laboratory of Veterinary Pharmaceutics Development and Safety Evaluation, South China Agricultural University, Guangzhou, China; ^3^Guangdong Laboratory for Lingnan Modern Agriculture, Guangzhou, China

**Keywords:** gamithromycin, PK/PD, *S. suis*, cutoff, piglet

## Abstract

Gamithromycin is a long-acting azalide antibiotic that has been developed recently for the treatment of swine respiratory diseases. In this study, the pharmacokinetic/pharmacodynamic (PK/PD) targets, PK/PD cutoff, and optimum dosing regimen of gamithromycin were evaluated in piglets against *Streptococcus suis* in China, including a subset with capsular serotype 2. Short post-antibiotic effects (PAEs) (0.5–2.6 h) and PA-SMEs (2.4–7.7 h) were observed for gamithromycin against *S. suis*. The serum matrix dramatically facilitated the intracellular uptake of gamithromycin by *S. suis* strains, thus contributing to the potentiation effect of serum on their susceptibilities, with a Mueller-Hinton broth (MHB)/serum minimum inhibitory concentration (MIC) ratio of 28.86 for *S. suis*. Dose-response relationship demonstrated the area under the concentration (AUC)/MIC ratio to be the predictive PK/PD index closely linked to activity (*R*^2^ > 0.93). For *S. suis* infections, the net stasis, 1–log_10_, and 2–log_10_ kill effects were achieved at serum AUC_24h_/MIC targets of 17.9, 49.1, and 166 h, respectively. At the current clinical dose of 6.0 mg/kg, gamithromycin PK/PD cutoff value was determined to be 8 mg/L. A PK/PD-based dose assessment demonstrated that the optimum dose regimen of gamithromycin to achieve effective treatments for the observed wild-type MIC distribution of *S. suis* in China with a probability of target attainment (PTA) ≥ 90% was 2.53 mg/kg in this study. These results will aid in the development of clinical dose-optimization studies and the establishment of clinical breakpoints for gamithromycin in the treatment of swine respiratory infections due to *S. suis*.

## Introduction

When considering swine respiratory disease (SRD) due to bacterial agents, the primary pathogens are *Actinobacillus pleuropneumoniae*, followed by *Bordetella bronchiseptica* and *Streptococcus suis* (1). Albeit relatively less frequent, the presence of *S. suis* capsular serotype 2, and a most virulent and dominant pathogenic serotype in many countries that have been associated with pneumonia, meningitis, and septicemia can result in severe systemic infections and high mortality in both pigs and human (2). In particular, two large outbreaks of lethal *S. suis* serotype 2 infections in China with 240 human cases and 53 deaths have changed the opinion that *S. suis* only causes sporadic human cases (3–5). Notwithstanding the existence of vaccination against streptococcosis, antibiotic therapy remains the most commonly used treatment for *S. suis* infections due to the serotype diversity.

Gamithromycin is a semisynthetic azalide that is approved for the treatment of SRD due to *A. pleuropneumoniae* and *Pasteurella multocida* (6, 7). Intramuscular and subcutaneous administrations have been developed with promising pharmacokinetic and efficacy results in pigs (8, 9). Our previous pharmacokinetic/pharmacodynamic (PK/PD) studies in piglets have demonstrated the potency and efficacy of gamithromycin for *Haemophilus parasuis* (6). Given that gamithromycin has activity against a wider range of respiratory pathogens and other macrolides/azalides are used for the treatment of respiratory tract infections (6, 10–12), a logical consideration is to what extent gamithromycin could be valuable for *S. suis* infections. While it is recognized that the area under the concentration-time curve/MIC (AUC/MIC) is the predictive PK/PD index that well-describes the efficacy of azalide antibiotics (9, 13–15), data regarding the PK/PD profiles of gamithromycin are not currently available for *S. suis*.

The objectives of this study were to characterize the PK/PD relationship and the magnitude of gamithromycin target exposures associated with efficacy against *S. suis*. Specifically, the PK/PD targets provide a framework for optimization of the dosing regimen of gamithromycin when combined with PK data from pigs. Moreover, by integration of these targets and the MIC distribution of *S. suis* isolates from China, the relevant PK/PD cutoff (CO_PD_) value for gamithromycin was estimated for *S. suis*. This would be useful in setting the final clinical susceptibility breakpoints for gamithromycin against *Streptococcus* spp.

## Materials and Methods

### Organisms, Media, and Antibiotic

A total of 197 clinical isolates of *S. suis* collected from diseased swine suffering septicemia, pneumonia, and meningitis were used in this study. These isolates were gathered through a 10-years antimicrobial resistance surveillance study for animal original bacteria in China between 2009 and 2018. Two well-described strains (ATCC 43765 and 05ZYH33) of *S. suis* serotype 2 (SS2) were used for the PK/PD studies, as SS2 is the most virulent and dominant pathogenic serotype for both swine and human (2, 16, 17). *S. suis* strains were grown, subcultured, and quantified using cation-adjusted Mueller-Hinton broth (MHB) with 5% lysed horse blood and sheep blood agar. Gamithromycin injection (Zactran^®^, 15% w/v) for *in vivo* studies was purchased from Boehringer Ingelheim Animal Health, Toulouse, France, and gamithromycin powder for *in vitro* studies was obtained from NMT Biotech Ltd., Suzhou, China.

### *In vitro* Susceptibility Testing and MIC Distribution

Routine MIC determinations for gamithromycin against 197 *S. suis* isolates were undertaken by the broth microdilution technique in accordance with CLSI guidelines (18). To obtain a wild-type MIC distribution, the isolates harboring the detectable acquired macrolide resistance mechanisms (i.e., *ermA, ermB, mefA*, and *msrD*) were consequently removed (19). Among the entire *S. suis* population, 47 representative isolates covering 10 different serotypes and 8 areas of China were selected for susceptibility measurements both in MHB and porcine serum to obtain a scaling factor that bridged the MICs in different matrices. To assess the extent of serum potentiation effect on gamithromycin susceptibility, further MIC determinations were, therefore, undertaken for additional five isolates of *S. suis*, in which MHB was supplemented with increasing proportions of porcine serum from 25 to 100%.

### Membrane Permeability Assessment

The plasma membrane permeability of *S. suis* cells in broth and serum matrices was measured using a 1-N-phenylnaphthylamine (NPN) uptake assay as previously established (20, 21). Bacterial cells from a mid-log phase culture were resuspended in PBS to an OD_600nm_ of 0.5 and inoculated into porcine serum and MHB supplemented with 25%, 50%, and 75% serum. After 4 h of incubation at 37°C, a volume of 100 μl of cells was added to 100 μl of PBS containing 25 μM NPN in black 96-well microplates and allowed to incubate for an additional 15 min at room temperature. Fluorescence was read in an EnSight plate reader (PerkinElmer; excitation λ = 355 nm, emission λ = 405 nm). NPN uptake results were calculated as a percentage of pure serum control.

### Intracellular Accumulation of Gamithromycin

To examine whether the serum matrix promotes drug transmembrane transport, the levels of gamithromycin intracellular accumulation by *S. suis* in MHB and porcine serum were determined as our previously established (21, 22). Bacterial cells from an overnight culture were adjusted in MHB and serum to a density of ~10^9^ CFU/ml and incubated for 30 min in the presence of 0.5 mg/L gamithromycin. After centrifugation, the cells were washed three times with PBS and lysed by sonication on ice. The gamithromycin concentrations in the lysate were determined using an HPLC-MS/MS system (9). Results are expressed as intracellular drug concentrations normalized to bacterial densities before lysis.

### *In vitro* Activity, PAE, and PA-SME Determinations

Time-kill experiments were performed to characterize *in vitro* activity of gamithromycin against *S. suis* as previously described (23). In brief, an initial inoculum of ~10^6^ CFU/ml mid-log phase *S. suis* cells was inoculated into MHB supplemented with serial concentrations of gamithromycin from 1/4 to 32 × MICs. After 3, 6, 9, 12, and 24 h of incubation, 10-fold serial dilutions of each culture were plated on blood agar for bacterial enumeration. The limit of detection was 40 CFU/ml.

The PAEs were measured after initial exposures to 1 × and 4 × MICs of gamithromycin, and the post-antibiotic sub-MIC effects (PA-SMEs) were determined in sub-MIC treated phases (0.1 to 0.3 × MICs) after exposure to gamithromycin at 4 × MICs for 1 h. The calculations of PAEs and PA-SMEs have been described in detail elsewhere (9, 23).

### Drug Pharmacokinetics and PK/PD Index Target Activity

Gamithromycin concentration measurement methods and serum PK in piglets after intramuscular injection at 6.0 mg/kg have been described elsewhere in detail (9). In brief, the elimination half-lives and the peak concentration of gamithromycin were 29.4 h and 0.99 mg/L, respectively (9).

The AUC/MIC ratio was chosen as the pharmacodynamic parameter for gamithromycin based on previous studies demonstrating this PK/PD index to be predictive of treatment efficacy for the macrolides (9, 23–25). *Ex vivo* PK/PD studies were performed using the drug-containing serums collected from piglets at different time points after receiving intramuscular dosing of gamithromycin at 6.0 mg/kg. Bacterial cells of ATCC 43,765 and 05ZYH33 were subcultured and inoculated to each serum sample, giving an initial inoculum of ~10^6^ CFU/ml. The mixtures were then serially diluted and plated using a drop-plate technique to measure the log_10_ change of bacterial count after 24 h of incubation. The PK/PD relationship was determined by measurement of the correlation between *ex vivo* activity and the PK/PD parameter of AUC_24h_/MIC. The dose-response curve was modeled according to a Hill-type sigmoid *E*_max_ model using the Phoenix WinNonlin software (version 8.1; Certara Corporation) (25): *E* = *E*_0_ + *E*_max_ × *C*^*N*^/(EC_50_^*N*^ + *C*^*N*^), where *E*_0_ is the log_10_ change of bacterial count in the absence of drug, *C* is the AUC_24h_/MIC ratio, EC_50_ is the AUC_24h_/MIC required to achieve 50% of the maximum effect (*E*_max_), and *N* is the Hill coefficient representing the slope of the dose-response curve. The coefficient of determination (*R*^2^) was used to estimate the variance that might be due to regression with the AUC_24h_/MIC ratio. The PK/PD index targets in serum necessary for the net static, 1-log_10_, and 2-log_10_ kill effects were determined for each strain.

### PK/PD-Based Dose Assessment and Cutoff Determination

To deduce the optimum dose of gamithromycin potentially for the treatment of *S. suis* infections, the population dose covering the observed wild-type MIC distribution of *S. suis* (*n* = 125) was explored by a 10,000-iterations Monte Carlo simulation (MCS) using the Crystal Ball software (Oracle, Redwood City, CA, United States). The wild-type distribution was chosen because only the isolates devoid of phenotypically detectable resistance genes that clinically can be successfully treated with gamithromycin and that should be considered to calculate a dose. In addition, macrolides generally act bacteriostatic effect (26). The population dose was, therefore, computed to guarantee a bacteriostatic activity over at least 3 days using the following formula (23, 25):


$$\begin{array}{l} \text{Dosage for }3\text{ days}\\ =\text{ MCS }\left(\frac{{\text{Cl}}_{3-\text{days}}\;\times \;{\text{AUC}/\text{MIC}}_{\text{breakpoint}}\;\times \;{\text{MIC}}_{\text{distribution}}\;}{\text{fu}\times \text{F}}\right) \end{array}$$

where Cl/*F* is the body clearance scaled by intramuscular bioavailability to cover at least 3 days dosing (L/kg/3 days) (9); AUC/MIC_breakpoint_ is the target PK/PD index of 17.9 h for a bacteriostatic effect; since a scaling factor of 28.86 was used to bridge the MIC variations between broth and serum that already took the serum binding into account, the free drug fraction (*fu*) was considered negligible herein.

For calculation of the PK/PD cutoff (CO_PD_), a series of MCSs were performed separately for each possible MIC based on our previously reported PK parameters and the PK/PD targets obtained in this study (9). The AUC_24h_/MIC ratio after intramuscular administration was calculated using the following equation: AUC_24h_/MIC = Dose/(Cl/*F* × MIC). Cl/*F* was the clearance scaled by intramuscular bioavailability that was assumed to be 0.91 ± 0.26 L/kg/h (9). The mean AUC_24h_/MIC ratio associated with a static endpoint (i.e., 17.9 h) was used for CO_PD_ determination. CO_PD_ was defined as the MIC value at which the probability of target attainment (PTA) reached 90% (27, 28).

## Results

### MIC Distribution for Gamithromycin

**Figure 1:**
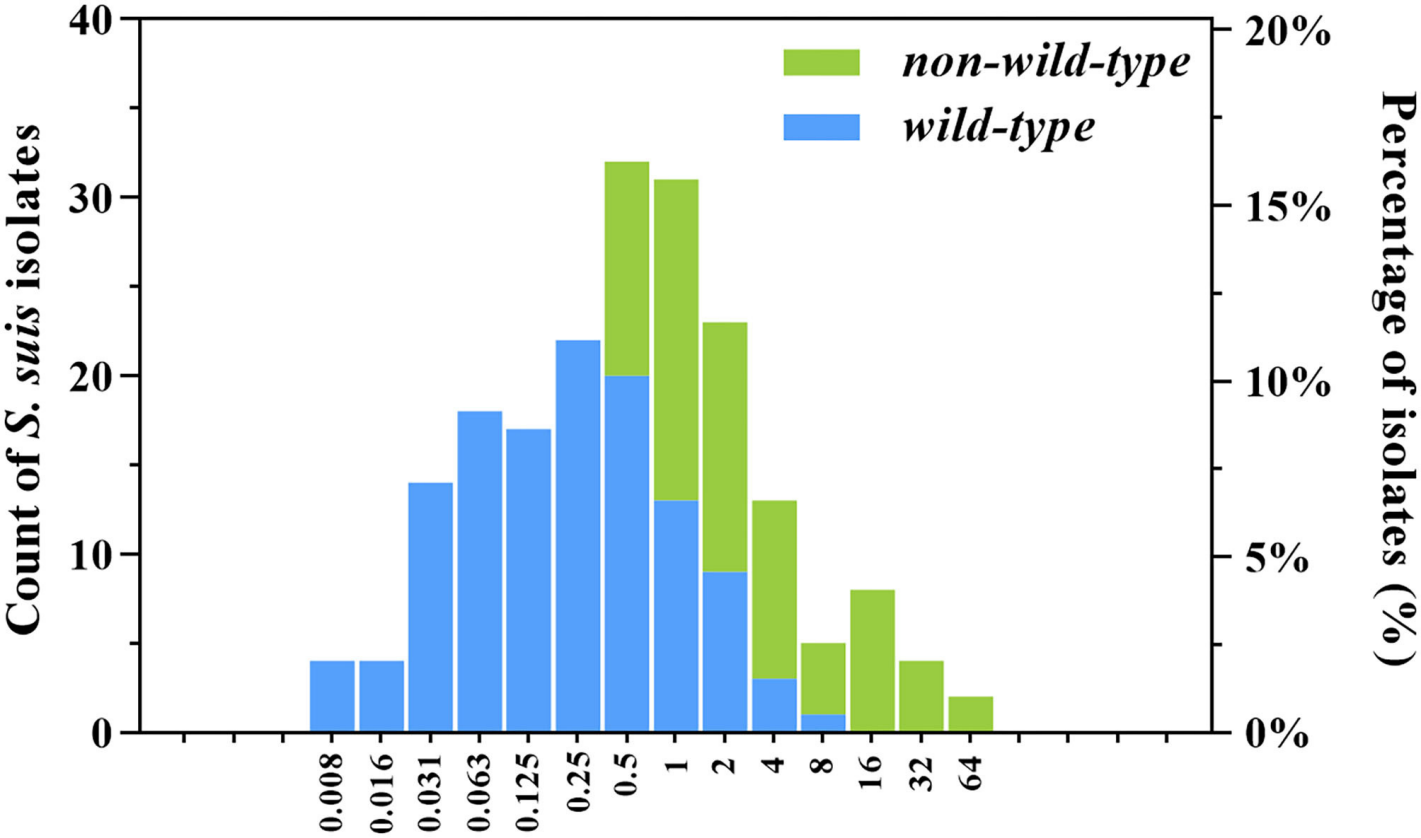
Wild-type and non-wild-type minimum inhibitory concentration (MIC) distributions for gamithromycin against clinical *Streptococcus suis* isolates from swine in China. The isolates harbored the macrolide resistance mechanisms such as *ermA, ermB, mefA*, and *msrD* genes (non-wild-type; *n* = 72) were consequently excluded from the primitive distribution (*n* = 197) to obtain the wild-type MIC distribution (*n* = 125). The number of *S. suis* isolates (left *y*-axis) and their observed frequency (right *y*-axis) corresponding to each MIC are shown along the *y*-axis.

As shown in the primitive gamithromycin MIC distribution for 197 *S. suis* isolates (Figure 1), the MIC_50_ and MIC_90_ values were 0.5 and 4 mg/L, respectively. After the removal of 72 isolates that carried *ermA, ermB, mefA*, or *msrD* genes, the other 125 *S. suis* isolates represented the phenotypical wild-type distribution (Figure 1). Based on the visual inspection and ECOFFinder method, the tentative wild-type cutoff was, therefore, set at 2 mg/L for the wild-type distribution obtained in this study.

### Possible Mechanisms for Serum-Induced Potentiation Effect

To characterize the impact of growth matrix on gamithromycin potency, we investigated the susceptibility discrepancy of *S. suis* between porcine serum and MHB. For the 47 representative *S. suis* isolates tested, geometric mean MIC and minimum bactericidal concentration (MBC) values were markedly lower in porcine serum than those determined in MHB, with an MHB/serum ratio of 28.86 for MIC and 11.23 for MBC, respectively (*P* < 0.005; paired Student's *t*-test; Table 1). Corresponding MBC/MIC ratios for both growth matrices were in the range of 3.45–8.87 (Table 1).

**Table 1:** Geometric mean minimum inhibitory concentrations (MICs) and minimum bactericidal concentrations (MBCs) in broth and porcine serum and MBC/MIC and Mueller-Hinton broth (MHB)/serum ratios for gamithromycin against selected *Streptococcus suis* isolates (*n* = 47).

**Test matrix**	**MIC (mg/L)**	**MBC (mg/L)**	**MBC/MIC ratio**
MHB	0.443	1.534	3.45
Serum	0.015	0.137	8.87
MHB/serum ratio^a^	28.86	11.23	NA

a *P <0.005 for MIC or MBC differences between MHB and serum (paired Student's t-test); NA, not applicable*.

**Figure 2:**
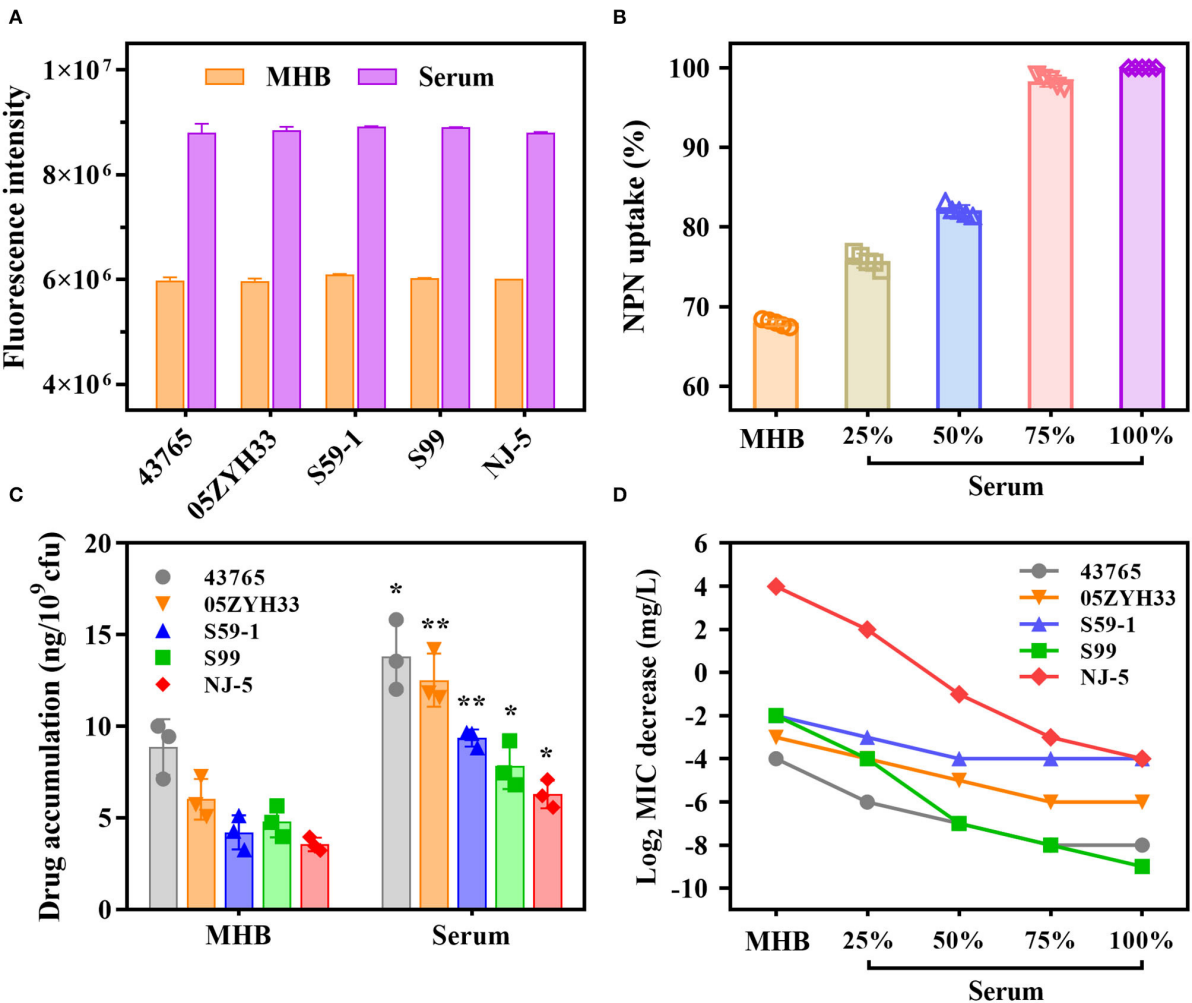
A large potentiation effect of serum on *in vitro* potency of gamithromycin against *S. suis*. **(A)** Porcine serum disrupts the plasma membrane of *S. suis* cells by measuring fluorescence intensity of 1-N-phenylnaphthylamine (NPN) after 4 h of incubation. **(B)** Serum-induced NPN uptake of *S. suis* strains. Bacterial cells were exposed to increasing proportions of porcine serum from 25 to 75%. NPN uptake (%) represents fluorescence intensity divided by the value observed in 100% serum. **(C)** Intracellular accumulations of gamithromycin in *S. suis* strains after exposure to 0.5 mg/L gamithromycin for 30 min in Mueller-Hinton broth (MHB) and serum matrix. Data shown are the means of three independent biological replicates, ^*^P < 0.05 and ^**^P < 0.005. **(D)** A concentration-dependent decrease in gamithromycin MICs for *S. suis* strains in the presence of increasing proportions of serum from 25 to 75%. Data shown are the log_2_-transformed MIC values.

1-N-Phenylnaphthylamine fluoresces strongly in phospholipid environments but weakly in aqueous environments. We observed that NPN fluorescence intensities for individual *S. suis* strains in serum were 1.5-fold greater compared with those in MHB (Figure 2A). The addition of varying proportions of porcine serum to MHB caused a dose-dependent increase in the NPN uptake (Figure 2B). Their results indicated that serum matrix markedly increased the plasma membrane permeability of *S. suis* cells, and therefore promoted the antibiotics intake. As expected, the intracellular accumulations of gamithromycin in *S. suis* cells are nearly doubled in serum matrix compared with MHB (*P* < 0.05; Figure 2C). Consistently, the MICs of gamithromycin against the five *S. suis* strains decreased exponentially with an increasing proportion of serum from 25 to 75% (Figure 2D).

### *In vitro* and *ex vivo* Activity of Gamithromycin for *S. suis* and PAEs

**Figure 3:**
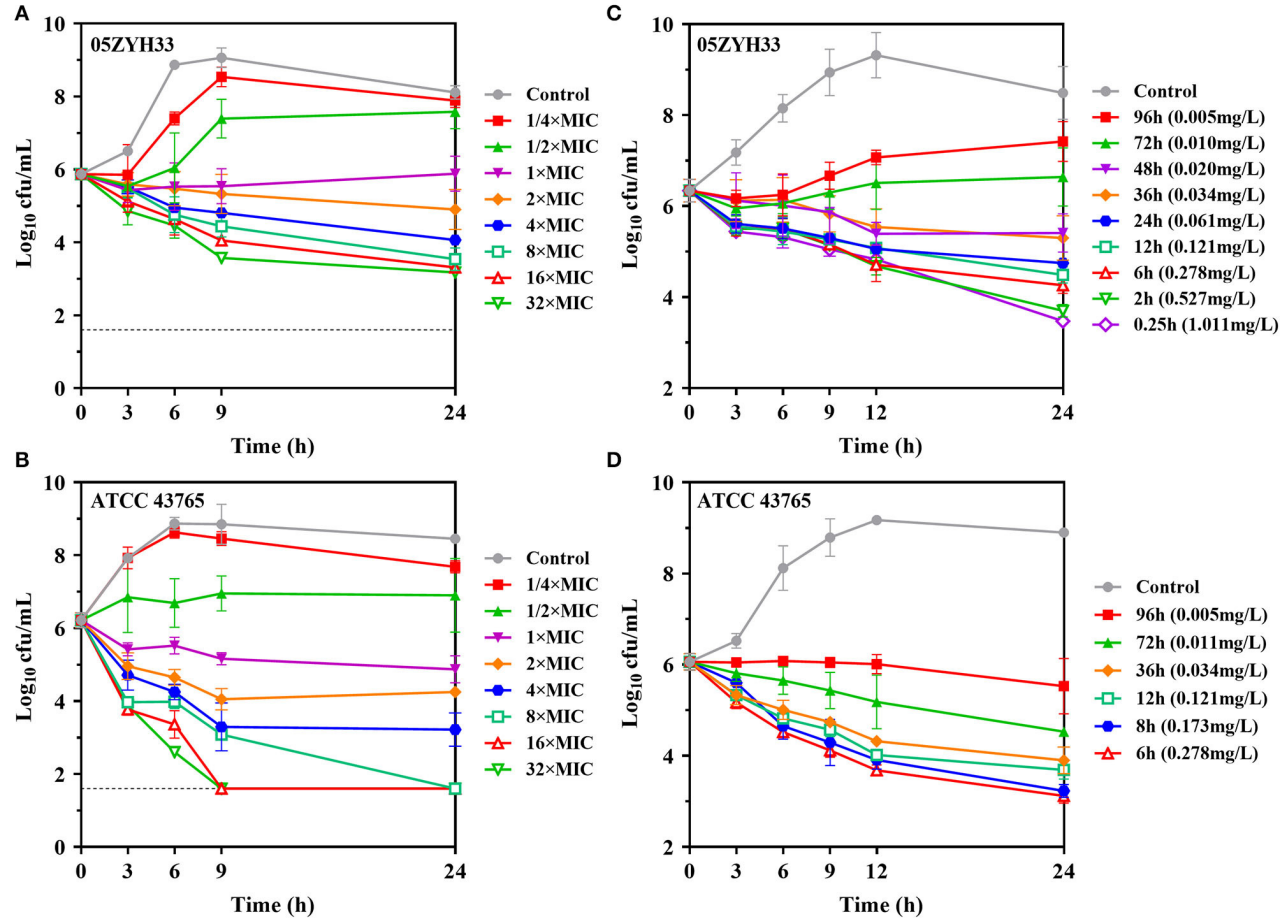
Gamithromycin activity against *S. suis*. **(A,B)** *In vitro* time-kill curves of gamithromycin against *S. suis* 05ZYH33 and ATCC 43765 in MH broth (MIC_MHB_ = 0.125 and 0.063 mg/L, respectively). **(C,D)** *Ex vivo* activity for *S. suis* 05ZYH33 and ATCC 43,765 in serum samples of piglets receiving intramuscular administration of gamithromycin at 6.0 mg/kg (MIC_serum_ = 0.016 and 0.004 mg/L, respectively). Numerical values on the right brackets represent the mean concentrations of gamithromycin in porcine serums collected at different time points.

*In vitro* time-kill curves of gamithromycin for *S. suis* were performed in the presence of 1/4 to 32 multiples of MIC. Gamithromycin exerted antibacterial activity immediately after being added to both *S. suis* isolates in a time-dependent manner (Figures 3A,B). Against *S. suis* ATCC 43,765, complete bactericidal activity reaching the undetectable limits of eradication was attained within 9 h in response to gamithromycin at 16 × MICs (Figure 3B).

Compared with the MHB matrix, gamithromycin exhibited higher *ex vivo* activity against *S. suis* in serum samples. For *S. suis* 05ZYH33, sustained killing was achieved with porcine serums obtained up to 36 h (>0.034 mg/L) after intramuscular injection at 6.0 mg/kg. Increasing the serum concentration of gamithromycin further to the peak concentration resulted in a decrease of 2.87-log_10_ CFU/ml after 24 h (Figure 3C). Persistent growth inhibition or rapid bactericidal activity was similarly observed for *S. suis* ATCC 43765 when exposed to serum collected up to 96 h (Figure 3D).

**Figure 4:**
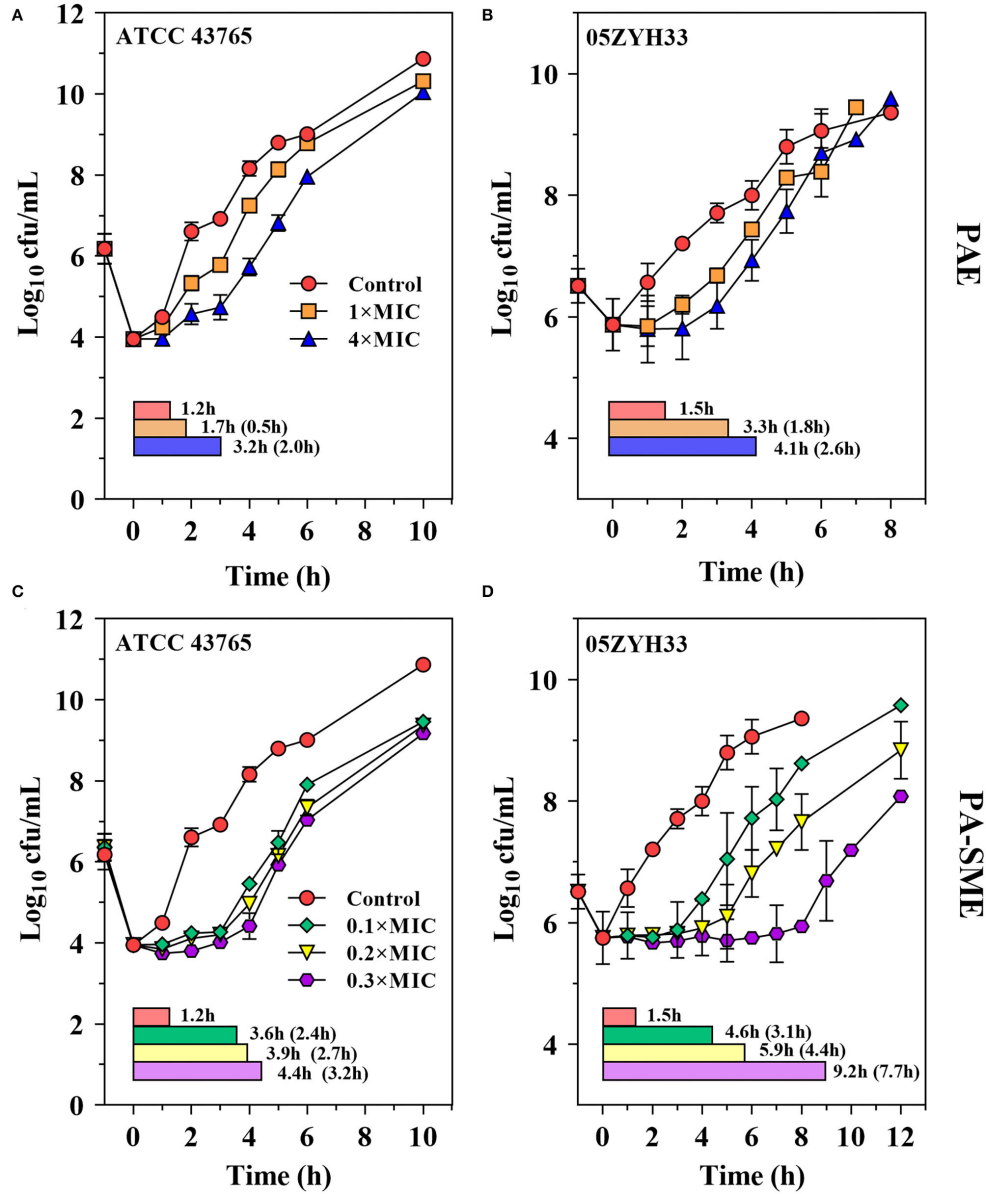
Gamithromycin postantibiotic effect [PAE; **(A,B)**] and post-antibiotic sub-MIC effect [PA-SME **(C,D**)] against *S. suis* ATCC 43765 and 05ZYH33. The post-antibiotic effects (PAEs) were measured after initial exposures to gamithromycin both at 1 × and 4 × MICs, and the PA-SMEs were measured after exposure to gamithromycin at 4 × MICs. The horizontal bars represent the time that required bacterial counts to increase by 1.0-log_10_ CFU/ml after drug removal (PAEs) or at the sub-MIC phases (PA-SMEs).

The PAEs for gamithromycin were calculated to be 0.5–1.8 h and 2.0–2.6 h at 1 × and 4 × MICs, respectively (Figures 4A,B). A concentration-dependent trend toward a longer suppression of *S. suis* regrowth was observed after exposure to the increasing sub-MIC concentrations of gamithromycin (0.1–0.3 × MICs), resulting in PA-SMEs of 2.4–7.7 h (Figures 4C,D). Of note, for gamithromycin having such a long half-life of 29.4 h (9), the short PAEs and PA-SMEs are of limited clinical significance for dosage determination and require dosage regimens that continuously maintain levels above the MIC.

### PK/PD Index Target of Gamithromycin for *S. suis*

**Figure 5:**
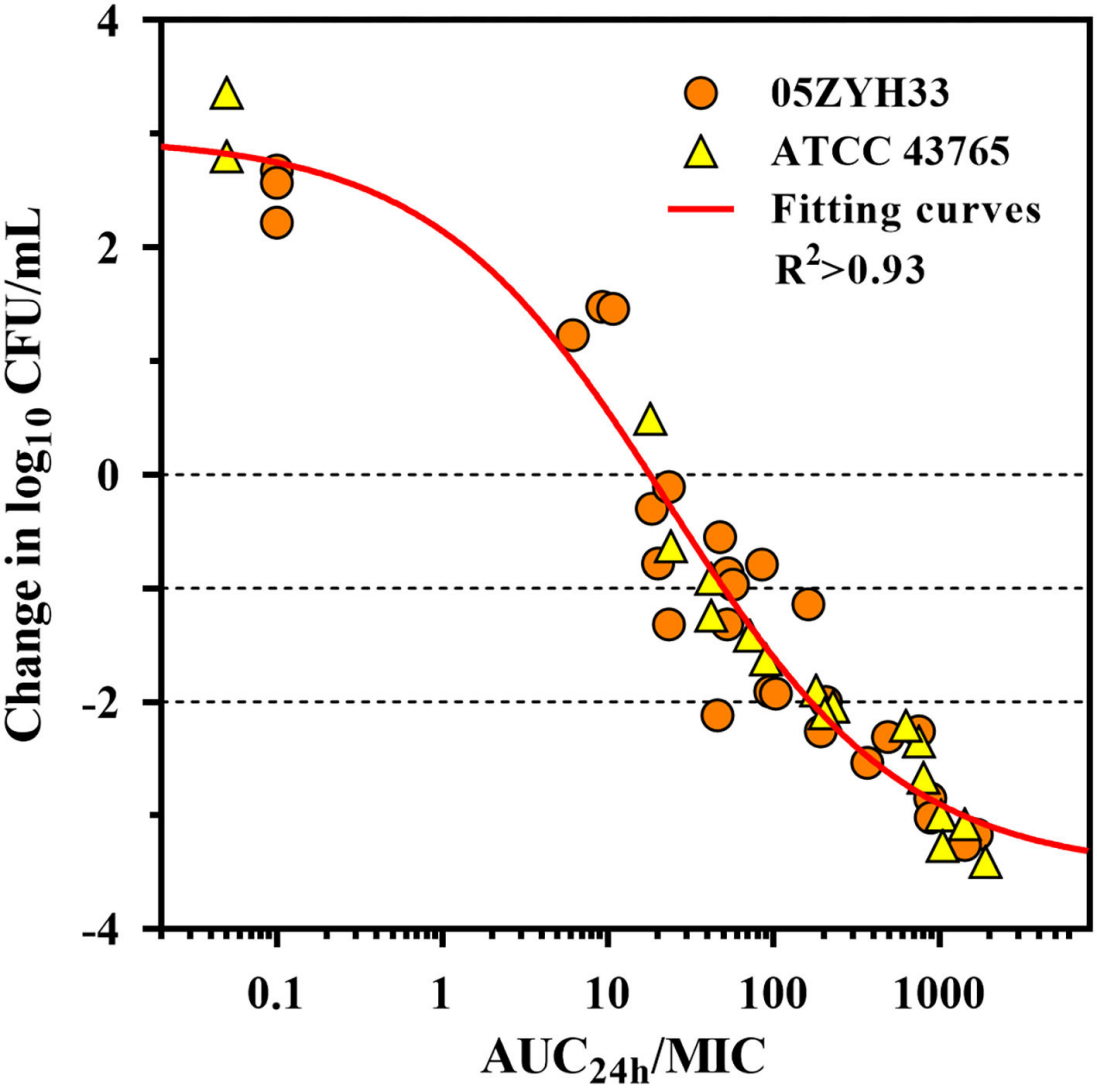
Gamithromycin pharmacokinetic/pharmacodynamic (PK/PD) relationship for *S. suis*. Correlation plots between *ex vivo* activity and area under the concentration (AUC_24h_)/MIC ratio of gamithromycin against *S. suis* 05ZYH33 and ATCC 43765 using the sigmoid *E*_max_ model. The fitting curve represents the predicted values, and the points represent the observed values of individual serum samples collected from 0 to 96 h.

The serum *in vitro* AUC_24h_/MIC is a robust predictor of the observed *in vitro* effect with an *R*^2^ value of >0.93 (Figure 5). The PK/PD relationships were quite similar for two strains (05ZYH33 and ATCC 43,765), which is reasonable given the relatively narrow MIC range. The mean AUC_24h_/MIC ratios necessary to produce the bacteriostatic effect, 1-log_10_ and 2-log_10_ kill effects in bacterial burdens were 17.9, 49.1, and 166 h, respectively, (Table 2). The mean AUC_24h_/MIC associated with a 1-log_10_ kill was roughly 3-fold larger than that associated with the stasis endpoint. The *E*_max_, EC_50_, and the slope (*N*) of the best-fitting line based on the sigmoid *E*_max_ model are also shown in Table 2. A bactericidal effect or greater kill was achieved for both *S. suis* strains tested over the entire drug-exposure range.

**Table 2:** Pharmacokinetic/pharmacodynamic (PK/PD) target values of gamithromycin in serum (AUC_24h_/MIC) needed to achieve the net stasis, 1-log_10_, and 2-log_10_ kill endpoints for each *S. suis* strain tested^a^.

***S. suis* strains**	**MIC (mg/L)**		**E_0_**	**E_max_**	**EC_50_**	**N**	**Target values of gamithromycin AUC_24h_/MIC ratio in serum (h)*^a^***		
	**in MHB**	**in serum**					**Stasis**	**1-log_**10**_ kill**	**2-log_**10**_ kill**
05ZYH33	0.125	0.016	2.70	−3.41	26.1	0.67	20.0	50.1	158
ATCC 43765	0.063	0.004	3.39	−3.71	19.2	0.51	15.8	48.0	174
Mean	NA	NA	3.05	−3.56	22.7	0.59	17.9	49.1	166
SD	NA	NA	0.35	0.15	3.45	0.08	2.10	1.05	7.85

a *E_0_, the change in bacterial density after 24 h of incubation in no drug control serum; EC_50_, the AUC_24h_/MIC ratio associated with 50% of the maximal bacterial reduction (E_max_); N, the slope of the dose-response relationship; means and standard deviations (SDs) are shown in boldface; NA, not applicable*.

### PK/PD-Based Dose Assessment and CO_PD_ Determination

**Figure 6:**
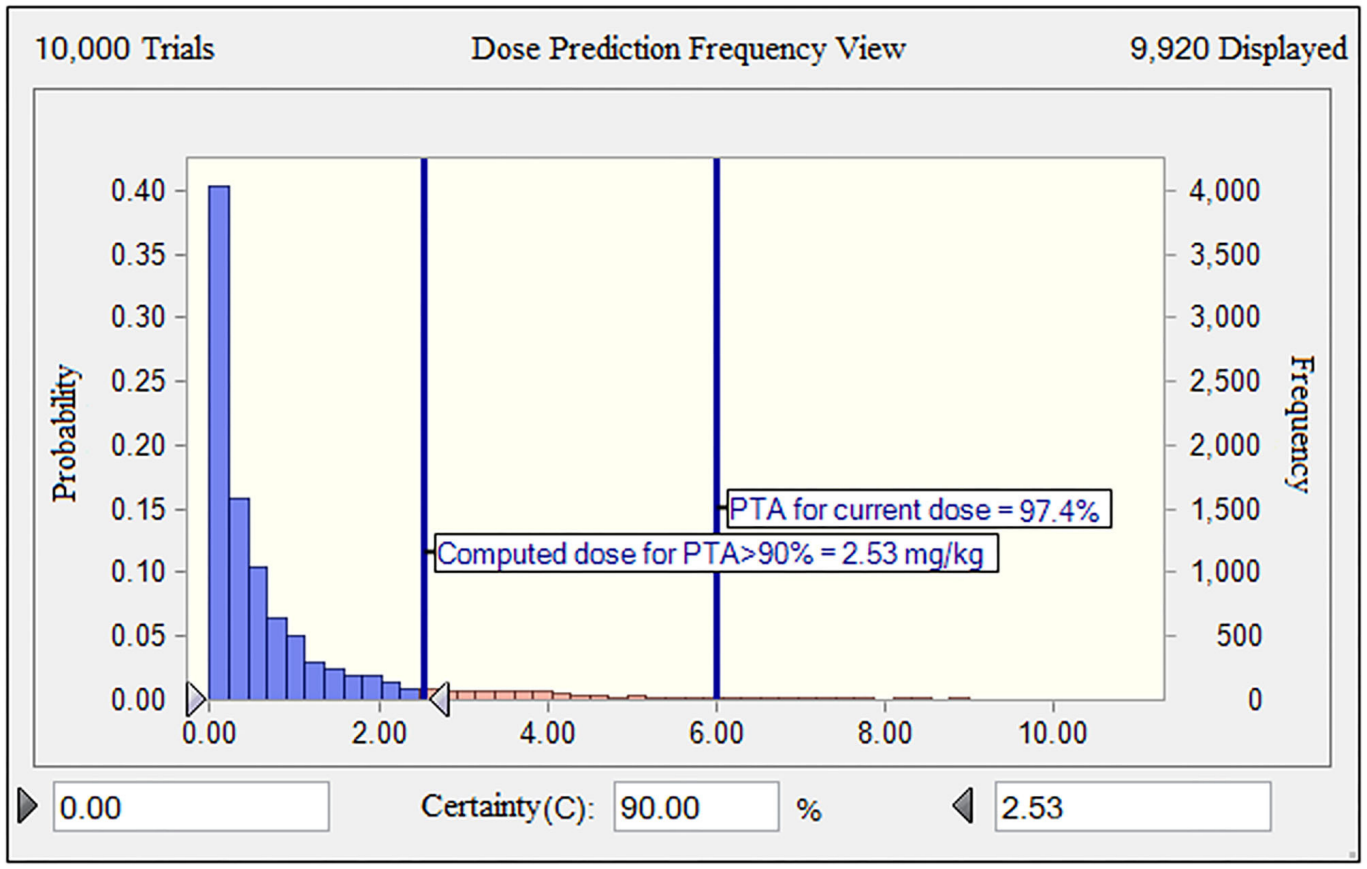
The population distributions of gamithromycin doses, as predicted by a PK/PD model against *S. suis* for a duration of action of 3 days in terms of probability of target attainment (PTA; *y*-axis). The vertical bars indicate the PTA (97.4%) for the current nominal dose of 6.0 mg/kg and the calculated target dose (2.53 mg/kg) for a PTA of >90% to cover the observed wild-type MIC distribution of *S. suis* obtained in this study. The dose range of 0–10 mg/kg was indicated on the x-axis.

Given the long half-life (29.4 h) of gamithromycin in piglets (9), the optimum dose should be administered to guarantee the activity of gamithromycin over at least 3 days for a bacteriostatic endpoint. The MHB MIC distribution was transformed into a vector of equivalent serum MIC distribution by dividing all determined MICs by 28.86, that is, by the scaling factor bridging MICs in the two matrices. Based on the results of MCS, for a PTA of 90%, the predicted dose for *S. suis* infections was 2.53 mg/kg in China (Figure 6). This suggested that the mean serum concentration of gamithromycin over 72 h after a single intramuscular administration of this dose would produce a net stasis for the observed wild-type MIC distribution of *S. suis* in China. For the current clinical dosage of 6.0 mg/kg, the corresponding PTA was 97.4% for this MIC distribution (Figure 6). In fact, this observed distribution of *S. suis* was collected from at least 8 different provinces of China through a 10-years antimicrobial resistance surveillance study. In this case, these isolates were typical enough to be representative of the *S. suis* population in China. Therefore, our dose prediction results based on MCS would be valid for treating SRD due to *S. suis* in China. However, given that the MIC distribution may vary widely between countries and regions, further evaluations are, therefore, needed to adjust the optimum dose according to susceptibility variation of different *S. suis* populations.

The PTA of achieving various AUC_24h_/MIC endpoints at each possible MIC value after a 10,000-subject MCS is shown in Table 3. At a MIC value of ≤ 8.0 mg/L, the probabilities of achieving a bacteriostatic action for *S. suis* (AUC_24h_/MIC ratio of 17.9 h) were higher than 86.2%, which is an approximation to the 90% criterion (Table 3). Consequently, the CO_PD_ of gamithromycin against *S. suis* was calculated as a MIC value of 8.0 mg/L.

**Table 3:** Probability of attaining the typical area under the concentration (AUC_24h_)/MIC targets at each possible MIC value when treated with gamithromycin at the current dose of 6.0 mg/kg against *S. suis* infections.

**Typical AUC_24h_/MIC targets (h)^a^**	**PTA (%) at each possible MIC (mg/L) in MH broth^a^**						
	**0.5**	**1**	**2**	**4**	**8**	**16**	**32**
17.9	100	100	100	99.7	86.2	12.3	1.03
49.1	100	100	99.9	45.7	3.53	0.42	0.06
166	100	69.4	6.41	0.51	0.12	0.02	0.01

a *A scaling factor of 28.86 was used to bridge the MIC differences between MH broth and serum when calculating the probabilities of target attainment (PTA); the mean AUC_24h_/MIC ratio in serum associated with a static endpoint (i.e., 17.9 h) was used for the PK/PD cutoff determination*.

## Discussion

In general, both the AUC/MIC and the time of plasma concentration above the MIC (T>MIC) are taken into account as the proper PK/PD parameters to determine the optimal dosing regimen of macrolides (13, 14). Contemporary thought on the long-acting (LA) macrolides in veterinary medicine are that the best PK/PD index is the AUC/MIC (15, 29). Our studies with gamithromycin have consistently demonstrated time-dependent activity against *S. suis* and *H. parasuis* with short PAEs and PA-SMEs (9). Data generated in this study identified the AUC/MIC as the PK/PD parameter that was strongly associated with the activity for *S. suis* with an *R*^2^ of >0.93. The mean serum AUC/MIC targets required for stasis, 1-log_10_, and 2-log_10_ reductions in this study were 17.9, 49.1, and 166 h, respectively. In our previous PK study where the clinical intramuscular dose of gamithromycin at 6.0 mg/kg was administered to piglets, the corresponding AUC value was 6.28 mg·h/L (9). After accounting for the scaling factor of 28.86 between MHB and serum, gamithromycin MIC_50_ and MIC_90_ values in serum for the observed distribution of *S. suis* in China would be 0.02 and 0.14 mg/L,respectively. Normalizing the AUC value for the serum MIC_50_ and MIC_90_ of gamithromycin achieved AUC/MIC ratios of 45 and 314 h, respectively, which would be adequate to exceed the stasis and 2-log_10_ reduction endpoints identified in this study.

The PK/PD target assessment for activity is a critical step in the development of antibiotic drugs to generate the optimal dosing regimens as well as establish the susceptibility breakpoints (30). Our studies demonstrated gamithromycin to be active against *S. suis* infections, while it is currently not indicated for the treatment of streptococcosis. As a result of its spectrum and potency against SRD-associated pathogens, the clinical efficacy of gamithromycin is currently being investigated in a confirmatory clinical trial with *S. suis* infections (7). From a microbiological success standpoint, gamithromycin at 6 mg/kg resulted in a 32.1% decrease in the bacterial isolation rate of *S. suis* from the porcine nasal cavity at the end of a single dose treatment, which was comparable to a 37.7% decrease in the tulathromycin treatment group (7). Consistent with our PK/PD-based dose simulation results in this study, the current clinical dose regimen of gamithromycin at 6.0 mg/kg is estimated to be effective against SRD due to *S. suis* in China.

Similar to other macrolides for fastidious bacteria such as *A. pleuropneumoniae, P. multocida*, and *H. parasuis* (31, 32), the MICs of gamithromycin for *S. suis* were substantially lower in porcine serum compared with those determined in MHB. It is well-known that macrolides act through binding to the 50S ribosomal subunit that requires drug across the plasma membrane of the bacterial cell first. This potentiation effect by serum is, therefore, likely due to increased plasma membrane permeability and subsequent passive accumulation of antibiotics in the biological matrix. In support of this hypothesis, our results from NPN uptake and intracellular antibiotic accumulation assays indicated that porcine serum significantly facilitated gamithromycin uptake by *S. suis* cells and reduced their MIC values in a concentration-dependent manner. This is in agreement with our previous studies for tulathromycin against *S. suis* and gamithromycin against *H. parasuis* (9, 23). These results also support the view that increased susceptibility to macrolides in eukaryotic cell culture media and biological fluids was due to the decreased expression of the *oprM* gene (encoding a protein essential for active multidrug efflux pumps MexAB and MexXY) in *Pseudomonas aeruginosa* (33). Collectively, these observations suggest that the clinical efficacy of macrolides may be largely underestimated due to the higher MICs measured in artificial media such as MHB.

Setting the conclusive epidemiological cutoff value for gamithromycin against *S. suis* was beyond the scope of this study, which would have required a larger population of phylogenetically diverse isolates from different laboratories and more extensive geographical origin (34, 35). In veterinary clinical settings, internationally accepted interpretative criteria are lacking for gamithromycin against *Streptococcus* spp. until now, but breakpoints of ≥16 mg/L have been proposed to classify cattle *P. multocida* and *Mannheimia haemolytica* isolates as resistant to gamithromycin (18). In this study, the PK/PD cutoff for gamithromycin against *S. suis* was calculated to be 8 mg/L at the current clinical dose of 6.0 mg/kg. This is ~2–4-fold higher than the MIC breakpoints for other macrolide antibiotics such as azithromycin, clarithromycin, and erythromycin against *Streptococcus* spp. recommended by CLSI guideline (18). In general, the PK/PD cutoff value was determined by considering the PK variability, microbiological, and pharmacological components. Of note, the PK variability was generally limited between different breeds of pigs. The PK/PD cutoff obtained in this study is, therefore, valid for countries other than China and can be taken into account by the CLSI and VetCAST to establish clinical breakpoints.

One of the major limitations of this study is the fact that only the serum PK/PD relationship was investigated. It will be important to test relevant PK/PD targets based on drug levels at the site of infections. In addition, our study was unable to establish the epidemiological cutoff solely based on the single MIC distribution of *S. suis*, and the optimum dose computations are somewhat restricted to China. Nonetheless, the PK/PD cutoff identified in this study can be used as a tentative surrogate for gamithromycin MIC interpretation against *S. suis* in the absence of a clinical breakpoint. Further investigations are required to confirm our findings and support the establishment of the final gamithromycin susceptibility breakpoints for *S. suis*.

## Conclusion

Our study demonstrated that serum exposure promotes intracellular uptake of gamithromycin by *S. suis* cells, contributing to the potentiation effect in the presence of porcine serum. The pharmacodynamic targets and PK/PD cutoff obtained in this study are of potential clinical relevance for the dosing regimen optimization and susceptibility breakpoint determination for gamithromycin in the treatment of SRD due to *S. suis*.

## Data Availability Statement

The raw data supporting the conclusions of this article will be made available by the authors, without undue reservation.

## Ethics Statement

The animal study was reviewed and approved by Institutional Animal Care and Use Committee (IACUC) of South China Agricultural University (SCAU).

## Author Contributions

Y-FZ designed the experiment and conceptualized the model. R-LW, and Y-FZ drafted the manuscript. R-LW, PL, X-FC, and XY carried out the experiments. X-PL revised the Introduction section. Y-HL and JS analyzed the data. All authors read and approved the final manuscript.

## Funding

This work was supported by the National Natural Science Foundation of China (31902318), the Foundation for Innovative Research Groups of the National Natural Science Foundation of China (32121004), the Local Innovative and Research Teams Project of Guangdong Pearl River Talents Program (2019BT02N054), Program for Changjiang Scholars and Innovative Research Team in University of Ministry of Education of China (IRT_17R39), Guangdong Major Project of Basic and Applied Basic Research (2020B0301030007), and the Innovative Team Project of Guangdong University (2019KCXTD001).

## Conflict of Interest

The authors declare that the research was conducted in the absence of any commercial or financial relationships that could be construed as a potential conflict of interest.

## Publisher's Note

All claims expressed in this article are solely those of the authors and do not necessarily represent those of their affiliated organizations, or those of the publisher, the editors and the reviewers. Any product that may be evaluated in this article, or claim that may be made by its manufacturer, is not guaranteed or endorsed by the publisher.

## References

[1] Sweeney MT Lindeman C Johansen L Mullins L Murray R Senn MK. Antimicrobial susceptibility of *Actinobacillus pleuropneumoniae, Pasteurella multocida, Streptococcus suis*, and *Bordetella bronchiseptica* isolated from pigs in the United States and Canada, 2011 to 2015. J Swine Health Prod. (2017) 25:106–20. Available online at: https://www.aasv.org/shap/issues/v25n3/v25n3p106.html (accessed June 28, 2022).

[2] Feng Y Zhang H Wu Z Wang S Cao M Hu D. Streptococcus suis infection: an emerging/reemerging challenge of bacterial infectious diseases? Virulence. (2014) 5:477–97. 10.4161/viru.28595 24667807 PMC4063810

[3] Zheng C Wei M Jia M Cao M. Involvement of various enzymes in the physiology and pathogenesis of *Streptococcus suis*. Vet Sci. (2020) 7:143. 10.3390/vetsci7040143 32977655 PMC7712317

[4] Tang J Wang C Feng Y Yang W Song H Chen Z. Streptococcal toxic shock syndrome caused by *Streptococcus suis* Serotype 2. PLoS MED. (2006) 3:e151. 10.1371/journal.pmed.0030151 16584289 PMC1434494

[5] Yu H Jing H Chen Z Zheng H Zhu X Wang H. Human *Streptococcus suis* Outbreak, Sichuan, China. Emerg Infect Dis. (2006) 12:914–20. 10.3201/eid1206.051194 16707046 PMC3373052

[6] Hamel D Richard-Mazet A Voisin F Bohne I Fraisse F Rauh R. Gamithromycin in swine: pharmacokinetics and clinical evaluation against swine respiratory disease. Vet Med Sci. (2021) 7:455–64. 10.1002/vms3.375 33058489 PMC8025653

[7] Xiao T Yang Y Zhang Y Cheng P Yu H Liu R. Efficacy of gamithromycin injection administered intramuscularly against bacterial swine respiratory disease. Res Vet Sci. (2020) 128:118–23. 10.1016/j.rvsc.2019.11.006 31778852

[8] Wyns H Meyer E Plessers E Watteyn A De Baere S De Backer P. Pharmacokinetics of gamithromycin after intravenous and subcutaneous administration in pigs. Res Vet Sci. (2014) 96:160–3. 10.1016/j.rvsc.2013.11.012 24331716

[9] Zhou YF Bu MX Liu P Sun J Liu YH Liao XP. Epidemiological and PK/PD cutoff values determination and PK/PD-based dose assessment of Gamithromycin against *Haemophilus parasuis* in piglets. BMC Vet Res. (2020) 16:81. 10.1186/s12917-020-02300-y 32138735 PMC7059257

[10] Shorr AF Zilberberg MD Kan J Hoffman J Micek ST Kollef MH. Azithromycin and survival in *Streptococcus pneumoniae* pneumonia: a retrospective study. BMJ Open. (2013) 3:e002898. 10.1136/bmjopen-2013-002898 23794577 PMC3686221

[11] Mitchell JD Goh S McKellar QA McKeever DJ. In Vitro pharmacodynamics of gamithromycin against *Mycoplasma mycoides* subspecies Mycoides small colony. Vet J. (2013) 197:806–11. 10.1016/j.tvjl.2013.05.025 23810743

[12] Baggott D Casartelli A Fraisse F Manavella C Marteau R Rehbein S. Demonstration of the metaphylactic use of gamithromycin against bacterial pathogens associated with bovine respiratory disease in a multicentre farm trial. Vet Rec. (2011) 168:241. 10.1136/vr.c6776 21493573 PMC3361959

[13] Craig WA. Pharmacokinetic/Pharmacodynamic parameters: rationale for antibacterial dosing of mice and men. Clin Infect Dis. (1998) 26:1–10. 10.1086/516284 9455502

[14] Watteyn A Devreese M De Baere S Wyns H Plessers E Boyen F. Pharmacokinetic and pharmacodynamic properties of gamithromycin in turkey poults with respect to *Ornithobacterium rhinotracheale*. Poult Sci. (2015) 94:2066–74. 10.3382/ps/pev217 26195808

[15] DeDonder KD Apley MD Li M Gehring R Harhay DM Lubbers BV. Pharmacokinetics and pharmacodynamics of gamithromycin in pulmonary epithelial lining fluid in naturally occurring bovine respiratory disease in multisource commingled feedlot cattle. J Vet Pharmacol Ther. (2016) 39:157–66. 10.1111/jvp.12267 26441021

[16] Lun ZR Wang QP Chen XG Li AX Zhu XQ. Streptococcus suis: an emerging zoonotic pathogen. Lancet Infect Dis. (2007) 7:201–9. 10.1016/S1473-3099(07)70001-4 17317601

[17] Chen C Tang J Dong W Wang C Feng Y Wang J. A glimpse of streptococcal toxic shock syndrome from comparative genomics of *S. suis* 2 Chinese isolates. PLoS ONE. (2007) 2:e315. 10.1371/journal.pone.0000315 17375201 PMC1820848

[18] CLSI. Performance Standards for Antimicrobial Disk and Dilution Susceptibility Tests for Bacteria Isolated From Animals: Approved Standard, 5th Ed. Wayne: CLSI Supplement Vet01S (2020).

[19] Zhang Y Tatsuno I Okada R Hata N Matsumoto M Isaka M. Predominant role of *msr(D)* over *mef(A)* in macrolide resistance in *Streptococcus pyogenes*. Microbiology. (2016) 162:46–52. 10.1099/mic.0.000206 26531240

[20] Saito H Sakakibara Y Sakata A Kurashige R Murakami D Kageshima H. Antibacterial activity of lysozyme-chitosan oligosaccharide conjugates (Lyzox) against *Pseudomonas aeruginosa, Acinetobacter baumannii* and methicillin-resistant *Staphylococcus aureus*. PLoS ONE. (2019) 14:e0217504. 10.1371/journal.pone.0217504 31136634 PMC6538184

[21] Zhou YF Liu P Zhang CJ Liao XP Sun J Liu YH. Colistin combined with tigecycline: a promising alternative strategy to combat *Escherichia coli* Harboring *bla* NDM-5 and *mcr-1*. Front Microbiol. (2020) 10:2957. 10.3389/fmicb.2019.02957 31969868 PMC6960404

[22] Chen Y Hu D Zhang Q Liao XP Liu YH Sun J. Efflux pump overexpression contributes to tigecycline heteroresistance in *Salmonella enterica, Serovar typhimurium*. Front Cell Infect Microbiol. (2017) 7:37. 10.3389/fcimb.2017.00037 28261566 PMC5313504

[23] Zhou YF Peng HM Bu MX Liu YH Sun J Liao XP. Pharmacodynamic evaluation and PK/PD-Based dose prediction of Tulathromycin: a potential new indication for *Streptococcus suis* infection. Front Pharmacol. (2017) 8:684. 10.3389/fphar.2017.00684 29033841 PMC5627010

[24] Guo LL Gao RY Wang LY Lin SJ Fang BH Zhao YD. In Vivo pharmacokinetic/pharmacodynamic (PK/PD) profiles of Tulathromycin in an experimental intraperitoneal *Haemophilus parasuis* infection model in Neutropenic Guinea Pigs. Front Vet Sci. (2021) 8:715887. 10.3389/fvets.2021.715887 34869712 PMC8632807

[25] Toutain PL Potter T Pelligand L Lacroix M Illambas J Lees P. Standard PK/PD concepts can be applied to determine a dosage regimen for a Macrolide: the case of Tulathromycin in the calf. J Vet Pharmacol Ther. (2017) 40:16–27. 10.1111/jvp.12333 27501187

[26] Watteyn A Plessers E Wyns H De Baere S De Backer P Croubels S. Pharmacokinetics of gamithromycin after intravenous and subcutaneous administration in broiler chickens. Poult Sci. (2013) 92:1516–22. 10.3382/ps.2012-02932 23687147

[27] Turnidge J Paterson DL. Setting and revising antibacterial susceptibility breakpoints. Clin Microbiol Rev. (2007) 20:391–408. 10.1128/CMR.00047-06 17630331 PMC1932754

[28] Tao MT Zhou YF Sun J Liu YH Liao XP. Establishment of valnemulin susceptibility breakpoint against *Clostridium perfringens* in rabbits. Anaerobe. (2017) 48:118–20. 10.1016/j.anaerobe.2017.08.006 28801120

[29] Toutain PL Pelligand L Lees P Bousquet-Melou A Ferran AA Turnidge JD. The Pharmacokinetic/Pharmacodynamic paradigm for antimicrobial drugs in veterinary medicine: recent advances and critical appraisal. J Vet Pharmacol Ther. (2021) 44:172–200. 10.1111/jvp.12917 33089523

[30] Lepak AJ Zhao M Marchillo K VanHecker J Andes DR. In Vivo pharmacodynamic evaluation of omadacycline (PTK 0796) against *Streptococcus pneumoniae* in the murine pneumonia model. Antimicrob Agents Chemother. (2017) 61:e02368–16. 10.1128/AAC.02368-16 28193651 PMC5404567

[31] Lees P Illambas J Potter TJ Pelligand L Rycroft A Toutain PL. large potentiation effect of serum on the *In Vitro* potency of tulathromycin against *Mannheimia haemolytica* and *Pasteurella multocida*. J Vet Pharmacol Ther. (2017) 40:419–28. 10.1111/jvp.12372 27891615

[32] Rose M Menge M Bohland C Zschiesche E Wilhelm C Kilp S. Pharmacokinetics of tildipirosin in porcine plasma, lung tissue, and bronchial fluid and effects of test conditions on *in Vitro* Activity against reference strains and field isolates of *Actinobacillus pleuropneumoniae*. J Vet Pharmacol Ther. (2013) 36:140–53. 10.1111/j.1365-2885.2012.01397.x 22500881

[33] Buyck JM Plesiat P Traore H Vanderbist F Tulkens PM Van Bambeke F. Increased susceptibility of *Pseudomonas aeruginosa* to macrolides and ketolides in eukaryotic cell culture media and biological fluids due to decreased expression of *OprM* and increased outer-membrane permeability. Clin Infect Dis. (2012) 55:534–42. 10.1093/cid/cis473 22573850

[34] Toutain PL Bousquet-Melou A Damborg P Ferran AA Mevius D Pelligand L. En route towards european clinical breakpoints for veterinary antimicrobial susceptibility testing: a position paper explaining the vetcast approach. Front Microbiol. (2017) 8:2344. 10.3389/fmicb.2017.02344 29326661 PMC5736858

[35] Yu X Wang G Chen S Wei G Shang Y Dong L. Wild-Type and Non-Wild-Type *Mycobacterium tuberculosis* MIC distributions for the novel fluoroquinolone antofloxacin compared with those for ofloxacin, levofloxacin, and moxifloxacin. Antimicrob Agents Chemother. (2016) 60:5232–7. 10.1128/AAC.00393-16 27324769 PMC4997829

